# Lemon peel essential oil and its nano-formulation to control *Agrotis ipsilon* (Lepidoptera: Noctuidae)

**DOI:** 10.1038/s41598-023-44670-x

**Published:** 2023-10-20

**Authors:** Habiba A. Ahmed, Amr A. Nassrallah, M. A. Abdel-Raheem, Huda H. Elbehery

**Affiliations:** 1 Plant Biochemistry Department, National Research Centre, Dokki, Giza 12622 Egypt; 2 Basic Applied Science Institute, Egypt-Japan University of Science and Technology (E-JUST), P.O. Box 179, New Borg El-Arab City, Alexandria 21934 Egypt; 3 Biochemistry Department, Faculty of Agriculture, Cairo University, Giza, 12613 Egypt; 4 Pests and Plant Protection Department, Agricultural and Biological Research Institute, National Research Centre, Dokki, Giza 12622 Egypt

**Keywords:** Biochemistry, Biotechnology, Plant sciences

## Abstract

Due to excessive use of synthetic pesticides the pest resistance developed along with pesticide residues accumulation in crops. Therefore, many nations are switching from chemical-based agriculture to “green” agriculture for pest control. The destructive pest black cutworm, *Agrotis ipsilon* (Hufnagel) (Lepidoptera: Noctuidae), is a polyphagous species that economically lead to extensive loss of a broad range of crops including corn, cotton, wheat, and many vegetables through the damage of foliar and roots. In this study, lemon peel essential oil (LPEO) was subjected to nano-formulation using polyethylene glycol as nanocarrier. The lethal activity of LPEO and its nano-form (LPEO-NPs) were tested against *A. ipsilon* second larval instar using feeding bioassay at different concentrations. Growth and developmental parameters, including larval and pupal duration, larval and pupal mortality, malformations % and adult emergence were evaluated. Results showed that LPEO exhibited insecticidal activity and causes different levels of effects on the development of *A. ipsilon* according to its concentration and formulation. In addition, at 75 mg/ml LPEO and LPEO-NPs significantly increased the larval mortality to 80.00% and 90.00%, respectively. The overall data revealed that insecticidal toxicity of LPEO was increased by nano-formulation.

Increasing food production is one of the main challenges to meet the global needs of the growing world population^1^. Unfortunately, agriculture crop loss caused by insect pests is considered one of the main causes for less intensive production^2^. *Agrotis ipsilon* (Hufnagel) (Lepidoptera: Noctuidae), black cutworm, is one of the extreme destructive pests that attack various crops such as corn, cotton, wheat, and many vegetables in many regions over the world. An extreme scale of economic loss through the destruction of plants foliar and roots. *A. ipsilon* moths deposit many hundred eggs on vegetation. once the eggs hatch, the early larval stages of *A. ipsilon* feed on the foliage of the crop. Therefore, the Black cutworm invasion caused significant yield reductions, especially at the seedling stage^3^. The current strategy for *A. ipsilon* control relies heavily on the use of several pesticides, which harm the environment and poses a threat to public health due to food accumulation and ground water or occasional exposure^4,5^. The need for efficient biodegradable and selective insecticides has increased due to the issues produced by these pesticides and their residues^6^. Therefore, to control pests, many nations are switching from chemical-based agriculture to “green” agriculture, which makes extensive use of biopesticides^7–9^. Novel strategies have included the innovation of new types of insecticides, and reevaluation and use of conventional pest control agents. Previous studies proved that pest communities have positive and negative interactions with a wide range of plants. Negative interaction with pests, which causes harm to the plants, has led to the development of various chemical defense mechanisms which considered good sources of new toxic compounds for pests^10–12^. Due to plant insect infection a broad range of secondary metabolites are produced to get adapted, either as part of their normal program of growth and development or in response to biotic stress^13^. Those secondary metabolites have toxic, repellent, and/or anti-nutritional effects on the pests^14^. Several organic compounds have been extracted from plants as volatile oils and other plant ingredients which show potential toxic effects against pest species^15^. The agro-industrial wastes such as Pomegranate Peels Extract and Orange Waste were used in pest control^7,16^. In this context, essential oil extracted from 4 citrus species displayed potent toxicity against *Callosobruchus maculatus* as a natural biopesticide^17,18^. In recent years, new technological approaches to overcome existing challenges in agriculture such as, lack in natural resources, and emergence of the new pests and climatic changes have been applied. Currently, researchers are developing traditional formulations to treat insects rely on high efficiency, through enhances solubility of insoluble active ingredients, ability for slow release, improved stability to avoid prematurely degradation, develop mobility and higher pesticidal activity. Nano-formulation is a new alternative strategy, utilizing natural products used as potential anti-insecticides due to physicochemical alteration, including size, shape, and charge^19^. Nanotechnology has become one of the most powerful technologies in the current years for managing many pests. This technology will lead to drastic changes in the agriculture field including future pest management. Over the past 20 years, various pesticide molecules offer a solution to problems like pesticide resistance and side effects have been approved of which 50% are NPs^20^. Nano formulations can solve specific agricultural issues due to their small size, high surface-area-to-volume ratio, and capacity to give unique crop protection approaches^21^. In addition, agricultural nanomaterials can play a high role in integrated pest management (IPM) programs through maintaining the number of pests below levels that may lead to economic losses with minimal impact on the environment^22^. In this regard, citrus essential oils as emulsions and included in polyethylene glycol (PEG) nanoparticles (EO-NPs exhibited a lethal against the invasive tomato *Tuta absoluta*^23^. Nano emulsions are used because they have greater stability compared to traditional emulsions. The advantages of the nano emulsions are prominent due to the small droplet size at the interface is highly significant. Due to its special physicochemical characteristics, nanotechnology is developing rapidly in many sectors, especially cosmetics, pharmaceutics, agriculture, and food industries^24^. Therefore, this study purposed to evaluate the insecticidal efficiency of lemon peel essential oil (LPEO) and Lemon peel Essential oil nanoparticles (LPEO-NPs) against *A. ipsilon* larvae.

## Material and methods

All chemicals used in this study were analytically grade purchased from Sigma Aldrich (USA).

### Essential oil extraction

Essential oil was extracted using cold pressing technique from dried pesticide-free certified Lemon peel obtained from El Marwa company, 6th October-Egypt.

### Nano-lemon essential oil preparation

Polyethylene glycol 4000 (PEG) was used in this study as nano carrier. In brief, 50 g of PEG 4000 was melted at 60 °C using hotplate stirrer, a mixture of 10 g of lemon essential oil dissolved into 2 ml of tween 80 was added dropwise to the melted PEG while stirring for 2 h at 15,000 rpm. The mixture was cooled at 4 °C for 24 h and ground in a refrigerated mortar. Finally, the product was sieved using a stainless-steel sieve (230 mesh), stored at room temperature in an airtight container^25,26^.

### Nano particles characterization.

#### Transmission electron microscopy (TEM)

The structure of the prepared nanoparticles was visualized using transmission electron microscopy (TEM) with a FEI TECNAI G2 F20 S-TWIN (Thermo Fisher Scientific, Waltham, MA, USA).

#### Fourier-transform infrared spectroscopy (FTIR)

The association with the nanocarrier and the functional groups were evaluated using Fourier-transform infrared spectroscopy (Bruker Optics Tensor 27, Bruker Corporation, Billerica, MA, USA).

#### Zeta Potential

The surface charges of the nanoparticles in water suspension at room temperature were determined by zeta potential Malvern Zeta Sizer (NanoZS, Malvern, UK).

### Gas chromatography-mass spectrometry

The GC–MS analysis of lemon essential oil was separated and identified using a Hewlett-Packard 5890 gas chromatograph, coupled to VG Analytical 70-250S mass spectrometer. The GC was equipped with a fused silica capillary CP-Sil 5 CB column (25 m × 0.25 mm i.d., film thickness 0.40 μm, from Chromback, Varian). Helium was used as carrier gas at a flow rate of 1 mL/min. The oven program started with an initial temperature of 80 °C held for 2 min and then the oven temperature was heated at 10 °C/min to 270 °C and finally held isothermally for 20 min. For GC–MS detection, an election ionization system, with ionization energy of 70 eV was used. A scan rate of 0.6 s (cycle time: 0.2 s) was applied, covering a mass range from 35 to 600 amu.

### Insect and plant rearing

Black Cutworm *A. ipsilon* larvae obtained from organic greenhouses of Faculty of Agriculture, Cairo University, Egypt. *A. ipsilon* were reared laboratory on castor plant leaves for many generations in the absence of pesticides inside polyester net cages (50 × 60 × 80 cm) at 25 ± 2 °C and 70–75% RH, with a photoperiod of 16:8 (L: D). Strips of black net were used as site of egg deposition. The newly deposited eggs were collected and placed in 1000 ml glass vials covered with muslin fixed tightly with a rubber band. The newly hatched larvae supplied daily with fresh castor leaves till the pupation. The pupae were incubated in glass jars until adult emergence and provided with pieces of cotton wool soaked in 10% honey solution as food supplement. The 2nd *A. ipsilon* larval instars of *S. frugiperda* were obtained from the colony for experiments.

### Toxicological bioassay

The examination toxicity of lemon peel essential oil (LPEO) and its nano-form (LPEO-NPs) on the second larval instar of *A. ipsilon* were executed using the leaf dipping method; where clean castor leaves were dipped for 20 s into LPEO or LPEO-NPs at different concentrations selected based on preliminary experiment (6.25, 11.63, 21.65, 40.30 and 75) dissolved in TWEEN 80 as emulsifier or deionized water, respectively. PEG and PEG-NPs particles alone at 75 mg/ml did not show any toxicity effects as compared to negative control (water and TWEEN 80+). All tested concentrations for toxicity study were performed in 3 replicates; each one had ten larvae of 2nd larval instar of *A. ipsilon*. The larvae were left to feed for one day on the treated leaf, and then, the larvae were provided daily with new untreated castor leaves. The experiments were examined daily to record the development and growth, including larval and pupal duration, larval and pupal mortality, percentage of malformations and adult emergence.

### Statistical analysis

The data were statistically analyzed using one-way ANOVA in SPSS computer program; means were compared using Duncan’s Multiple Range Test at the 0.05 level of probability^27^.

### Ethics approval and consent to participate

This experiment does not involve human experiments and animal experiments. The use of black cutworm, *Agrotis ipsilon* (Hufnagel) (Lepidoptera: Noctuidae) was supervised by prof. Abdel-Raheem M. A. All methods were performed in accordance with the relevant guidelines and regulations.

## Results

### Nano-characterization

**Figure 1:**
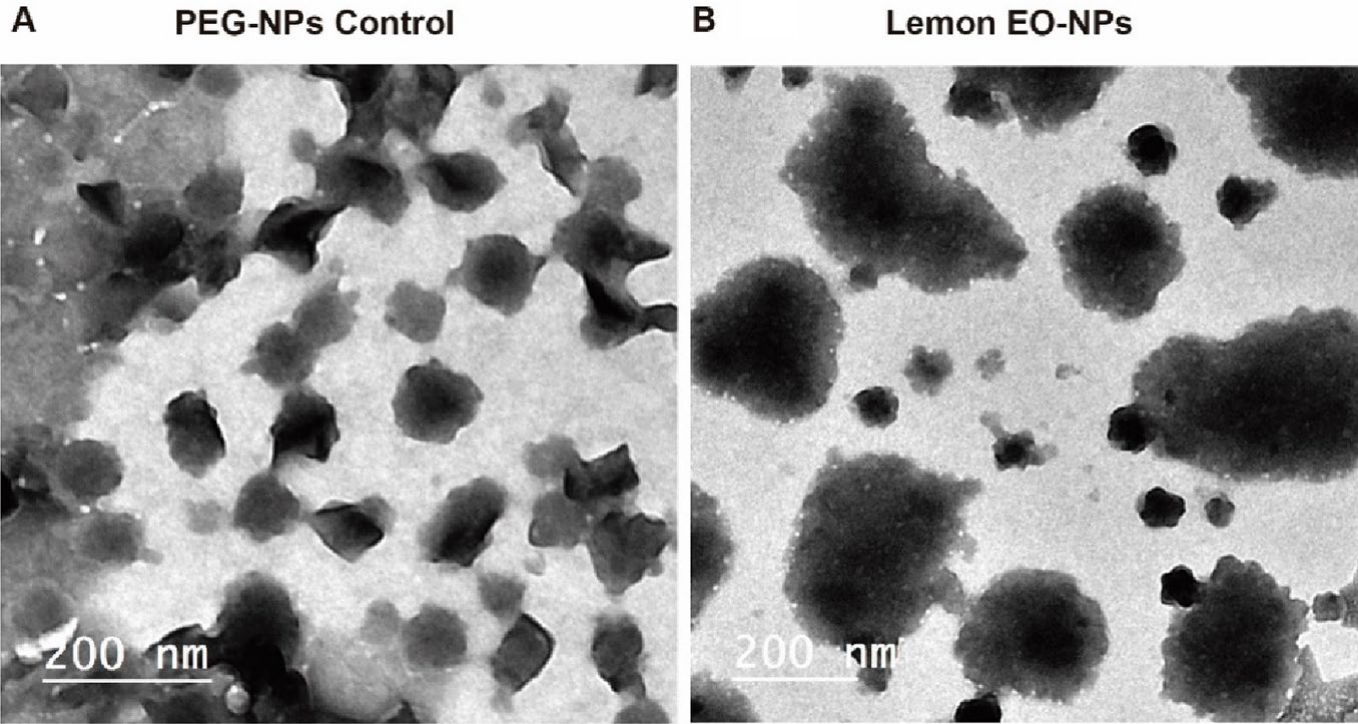
TEM images for nanoformulations of PEGNPs control (**A**) TEM image: LPEO-NPs (**B**).

Lemon peel essential oil loaded into polyethylene glycol (PEG) was subjected to physical characterization. Various techniques covering the main techniques used in nanoparticles characterization were used including TEM, FTIR and zeta potential. The results of TEM indicated that the generated LPEO-NPs were in nano-size when the size of the particles falls within the sub-micrometer range compared to respective controls Fig. 1. Moreover, the TEM observations confirmed that the size LPEO-NPs consisting of clusters of spherical properties approximately 75 nm of size.

### FTIR characterization

**Figure 2:**
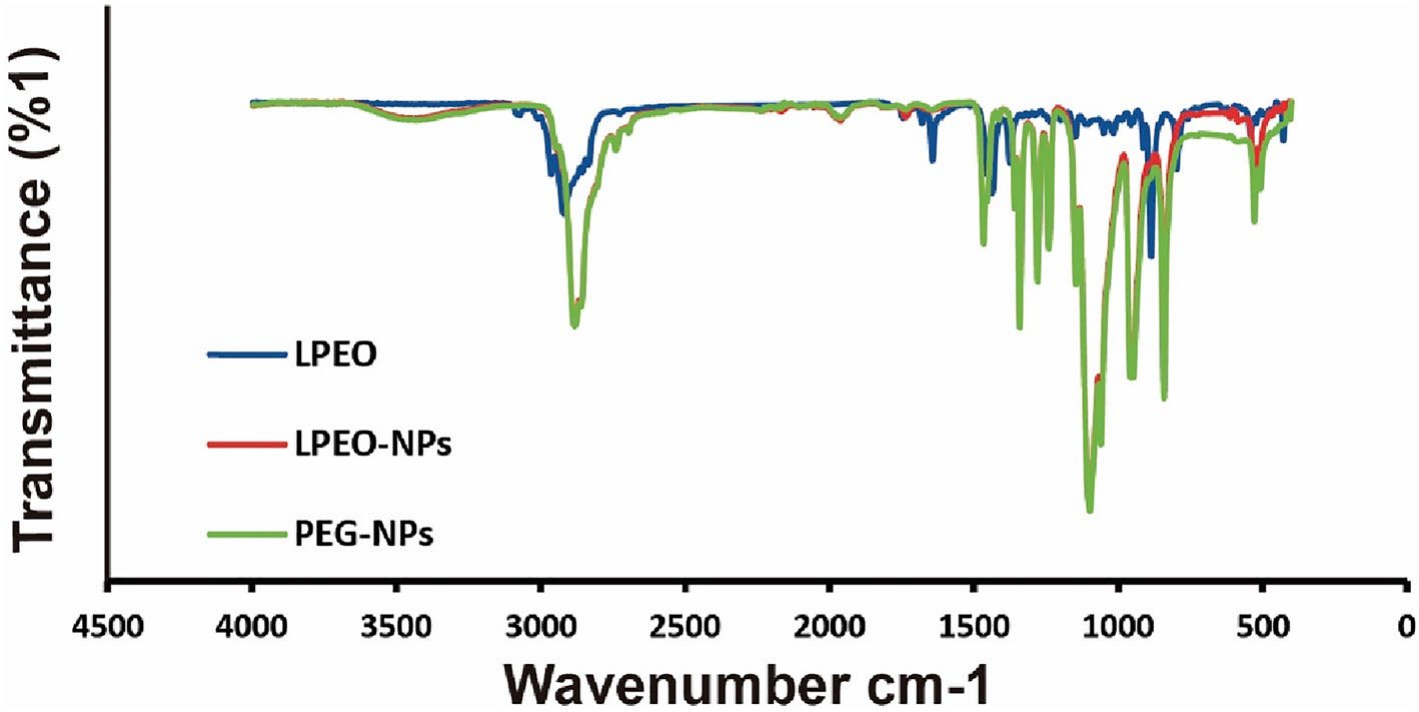
FTIR spectra of prepared nanoparticles. LPEO in red, LPEO-NPs in blue and PEG-NPs in black.

FTIR spectrum showed slight changes in surface-modified nanocarrier and nano-formulations (Fig. 2). In LPEO-NPs spectrum showed a clearly two peaks were shifted at 405.75 and 2164.45 cm^−1^, a new peak at 611.69 cm^−1^, which strongly confirmed the surface modification compared with PEG-NPs control. These peaks could have corresponded to the presence of loaded of LPEO.

### Zeta Potential Measurement

**Figure 3:**
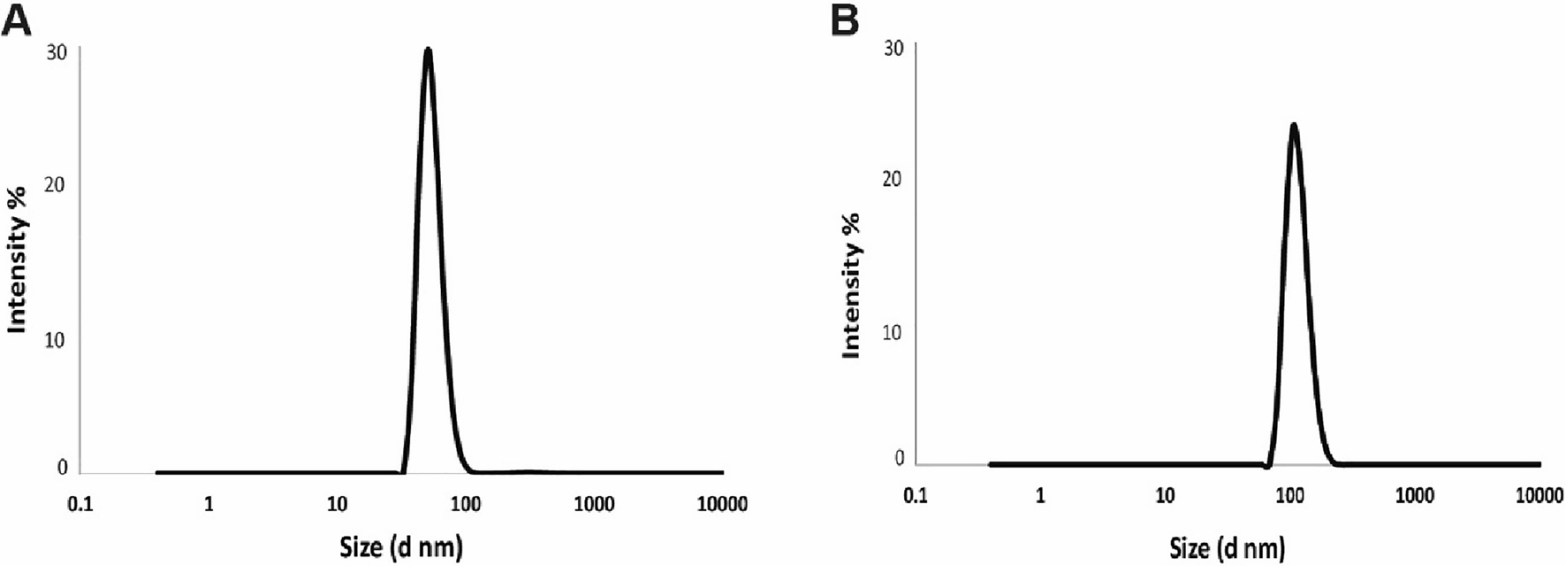
Particale size measurement of PEG-NPs (**A**) and LPEO-NPs (**B**) suspended in deionized water.

The changes in zeta potential values for LPEO-NPs compared to PEG-NPs were shown in Fig. 3 and Table 1. The results indicated that both PEG-NPs and LPEO-NPs showed negative surface charge in deionized water of − 10.1 ± 0.5 mV and − 11.5 ± 0.9 mV, respectively. As well as the poly dispersed index PDI value was 0.447 and 0.355, respectively indicated the homogeneity of the formulations. These results indicate that our nano-formulation lemon peel essential oil was nano size.

**Table 1:** Zeta potential and PDI of produced nano-essential oil.

Parameter	PEG-NPs control	Lemon EO-NPs
Zeta potential	− 10.1	− 11.5
PDI	0.447	0.355

### Lemon essential oil composition

The composition of the essential oil extracted from lemon peel was determined using GC–MS analysis. As shown in Table 2, GC–MS analysis identified 13 different compounds in the essential oil extracted from lemon peel representing 100% of total oil. D-Limonene was the most abundant compound with 57.65%, followed by γ-Terpinene with 14.45% and β-Pinene with 11.43% of total compounds. Anyway, several papers have reported that essential oil of lemon peel composition showed high variation between cultivar and growing place^28^.

**Table 2:** Lemon peel essential oil composition.

Compound	RT	Area Sum %
β-Thujene	6.189	0.91
α-Pinene	6.366	3.49
Sabinene	7.333	3.01
β-Pinene	7.43	11.43
β-Myrcene	7.745	2.66
D-Limonene	8.844	57.65
γ-Terpinene	9.605	14.45
Terpinolene	10.417	0.94
β-Citral	14.674	1
α-Citral	15.47	1.57
Neryl acetate	17.942	0.67
cis-α-Bergamotene	19.83	0.94
β-Bisabolene	21.627	1.27

### Toxicological bioassay

**Figure 4:**
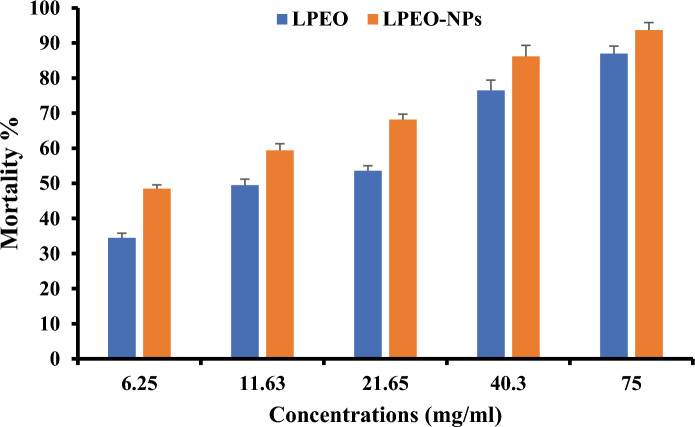
Percentage Accumulated mortality of A. ipsilon treated with different concentrations of LPEO and LPEO-NPs.

The results indicated that high concentrations of essential oil and nano essential oil (75 mg/mL) caused 86.67 + 8.82% mortality, while the LPEO-NPs exhibited 93.33 + 3.33% Fig. 4. The toxicity effect of LPEO and LPEO-NPs against the 2nd instars larvae of *A. ipsilon* were shown in Table 3. As a result, an acute toxic effect at larvae and adult stages of *A. ipsilon* was observed due to LPEO and LPEO-NPs treatment. In addition, significant toxic effects were obtained in response to higher concentrations and significantly prolonged the duration of larvae compared to the control. Furthermore, both LPEO and LPEO-NPs treatment significantly increased the larval duration at 75 mg/ml with a duration period of 21.25 ± 0.48 and 23.6 ± 0.51 days, respectively. However, the larval duration at 6.25 mg/ml was 17.81 ± 0.26 and 18.40 ± 0.52 days compared to 16.63 ± 0.38 days for control. Additionally, pupal duration was extremely longer when larvae were fed on castor leaves treated with high concentrations (75 mg/mL) of LPEO and LPEO-NPs compared to negative control with the duration period of 19.50 ± 0.29, 22.30 ± 0.37 and 10.63 days, respectively. Accordingly, the LPEO and LPEO-NPs led to a delayed effect on the larvae formation via reducing the number of emerged larvae that reached adulthood by treatment with the highest concentration of LPEO-NPs 75 mg/ml compared to control, therefore confirming their disruptive effect on insect growth Table 3. Adult Emergence % declined to (13.33 ± 8.2% and 6.67 ± 3.33%) at high concentration (75 mg/ml) of LPEO and LPEO-NPs respectively. All adult emerged from LPEO-NPs treatment were malformed and died. The surviving larvae fed on castor leaves treated with 21.63 mg/ml of LPEO developed into pupae. However, among the 63.33% emerged adults, 16.67% exhibited deformations properties.

**Table 3:** Effect of LPEO and LPEO-NPs on some development parameters of *Agrotis ipsilon.*

Treatment	Concentration (mg/ml)	Larval duration	Pupal duration	Adult emergence %
		Mean ± S.E		
LPEO	75	21.25 ± 0.48b	19.50 ± 0.29c	13.33 ± 8.82ef
	40.3	20.89 ± 0.31b	18.89 ± 0.26cd	23.33 ± 8.82def
	21.65	19.67 ± 0.37cd	18.67 ± 0.37cd	63.33 ± 14.53b
	11.63	18.73 ± 0.27def	18.09 ± 0.28d	46.67 ± 3.33bcd
	6.25	17.82 ± 0.26f.	18.18 ± 0.30d	56.67 ± 8.82bc
LPEO-NP	75	23.60 ± 0.51a	22.80 ± 0.37a	6.67 ± 3.33f.
	40.3	21.38 ± 0.46b	21.50 ± 0.42b	13.33 ± 3.33ef
	21.65	20.38 ± 0.38bc	19.63 ± 0.46c	30.00 ± 10.00cdef
	11.63	19.09 ± 0.28de	19.18 ± 0.18cd	40.00 ± 20.82bcde
	6.25	18.40 ± 0.52ef	18.90 ± 0.31cd	53.33 ± 3.33bcd
Control	0.00	16.63 ± 0.38g	10.63 ± 0.38e	100.00 ± 0.00a
	F	21.721**	71.58**	8.13**
	Sig	0.00	0.00	0.00

Means in a Column followed with the same letter(s) are not significantly different at 5% level of probability.

**Highly significant.

The mortality of the larval and pupae stage *A. ipsilon* was shown in Table 4. In this context, nano-formulations of LPEO significantly increased the larval mortality to 90.00%, while LPEO treatment displayed 80.00% at 75 mg/ml. However, no significant larval mortality was observed in negative control. Although we observed that larvae mortality % were concentration dependent at higher concentrations 21.63, 40.3 and75 mg/ml, the pupal mortality was 10.0, 20.0 and 6.67% respectively in LPEO treatment and 40, 30 and 3.33% in the case of LPEO-NPs. Surprisingly, lower concentration at 6.25 and 11.63 mg/ml showed higher pupal mortality compared to higher concentration with pupal mortality of 26.67 and 33.33% in response to LPEO while LPEO-NPs exhibited 30 and 43.33%, respectively Table 4. These results can be explained by the accumulative resistance due to high dose treatment. In this regard, we cannot exclude alternative cytotoxic mechanisms and targeted key elements including penetration level and defense mechanism. Based on the findings, the highest concentration of the tested oil caused a high level of larval mortality, and we did not record any larval malformations at 75 mg/ml Table 5.

**Table 4:** Percentage Larval and Pupal mortality of *Agrotis ipsilon* treated with LPEO and LPEO-NP.

Treatment	Concentration (mg/ml)	Larval mortality %	Pupal mortality %
		Mean ± SE	
LPEO	75	80.00 ± 15.28^a^	6.67 ± 6.67^cd^
	40.3	56.67 ± 17.64^ab^	20.00 ± 10.00^abcd^
	21.65	26.67 ± 8.82^bc^	10.00 ± 5.77^bcd^
	11.63	20.00 ± 10.00^c^	33.33 ± 12.02^ab^
	6.25	16.67 ± 8.82^c^	26.67 ± 8.82^abc^
LPEO-NP	75	90.00 ± 0.00^a^	3.33 ± 3.33^d^
	40.3	56.67 ± 8.82^ab^	30.00 ± 10.00^abc^
	21.65	30.00 ± 10.00^bc^	40.00 ± 0.00^a^
	11.63	30.00 ± 17.32^bc^	30.00 ± 5.77^abc^
	6.25	3.33 ± 3.33^c^	43.33 ± 3.33^a^
Control	0.00	0.00 ± 0.00^c^	0.00 ± 0.00^d^
	F	7.54**	4.50**
	Sig	0.00	0.00

Means in a Column followed with the same letter(s) are not significantly different at 5% level of probability.

**Highly significant.

**Table 5:** Malformations % of Larval, pupal, and Adult of *Agrotis ipsilon* produced by treatment with different concentrations of LPEO and LPEO-NP.

Treatment	Concentration (mg/ml)	Larval Malformation %	Pupal malformation %	Adult malformation %	Total malformation %
		Mean ± SE			
LPEO	75	0.00 ± 0.00a	0.00 ± 0.00c	0.00 ± 0.00c	0.00 ± 0.00d
	40.3	0.00 ± 0.00a	0.00 ± 0.00c	0.00 ± 0.00c	0.00 ± 0.00d
	21.65	3.33 ± 3.33a	6.67 ± 3.33abc	16.67 ± 12.02ab	26.67 ± 6.67abc
	11.63	0.00 ± 0.00a	6.67 ± 3.33abc	3.33 ± 3.33bc	10.00 ± 0.00cd
	6.25	3.33 ± 3.33a	6.67 ± 3.33abc	0.00 ± 0.00c	10.00 ± 0.00cd
LPEO-NP	75	0.00 ± 0.00a	0.00 ± 0.00c	6.67 ± 3.33bc	6.67 ± 3.33d
	40.3	3.33 ± 3.33a	16.67 ± 3.33a	10.00 ± 0.00abc	30.00 ± 5.77ab
	21.65	3.33 ± 3.33a	3.33 ± 3.33bc	0.00 ± 0.00c	6.67 ± 3.33d
	11.63	0.00 ± 0.00a	13.33 ± 8.82ab	3.33 ± 3.33bc	16.67 ± 12.02bcd
	6.25	3.33 ± 3.33a	13.33 ± 3.33ab	23.33 ± 6.67a	40.00 ± 11.55a
Control	0.00	0.00 ± 0.00a	0.00 ± 0.00c	0.00 ± 0.00c	0.00 ± 0.00d
	F	0.60ns	2.85*	3.09*	5.31**
	Sig	0.80	0.02	0.01	0.00

Means in a Column followed with the same letter(s) are not significantly different at 5% level of probability.

**Highly significant, *significant, ns = non-significant.

**Figure 5:**
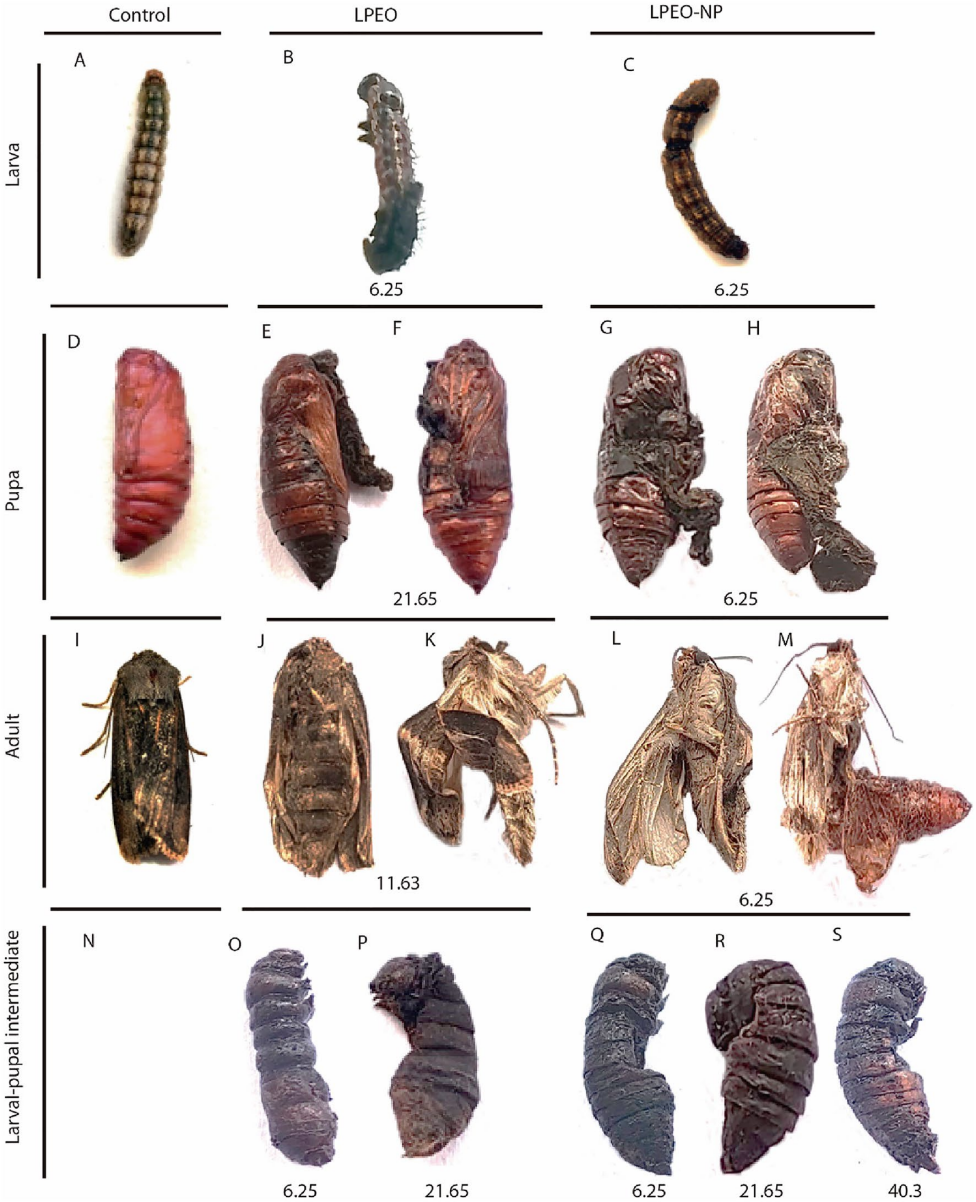
Normal stages (**A**, **D**, **I**) and Various stages malformations of *Agrotis ipsilon* treated with different concentrations of LPEO and LPEO-NP at different concentrations.

The finding exhibited that the treatment with essential oil showed insect growth regulatory activity against *A. ipsilon* larvae and produced several abnormal larvae, pupae, and adults Fig. 5. Whereas both LPEO and LPEO-NPs at 6.25 mg/ml does not allow larval to completely molting as shown in Fig. 5B,C compared to untreated larval Fig. 5A,D or formed Larval-pupal intermediates as shown in Fig. 5N,O in LPEO and Fig. 5P–R in LPEO-NPs compared to untreated larval Fig. 5A,D, respectively. Although, some of the larvae succeed to the pupae stage but it failed to get emerging. The symptoms of pupa failure were observed as showed in Fig. 5F,E and G,H failed to get rid of their larval or pupal molted skin at 2.5 mg/ml LPEO and 11.63 mg/ml LPEO-NPs, respectively. Some of the emerged adults were had some deformations, there were deformed (folded) wings from the adults that emerged from the cutworm treated with 12.5 mg/ml LPEO and 6.25 mg/ml LPEO-NPs Fig. 5K,L while other adult failed to get rid pupal skin Fig. 5M. Fig. 5J,K and L,M at 11.63 mg/ml LPEO and 6.25 mg/ml LPEO-NPs, respectively compared to normal adult Fig. 5I. As shown in Table 5 showed, the highest percentage of adult and total malformation was obtained at 6.25 mg/ml of LPEO-NP with 23.33 ± 6.67% and 40 + 11.55%, respectively.

## Discussion

Agro-waste-derived material converted into valuable nano-product recruiting researchers as feasible alternative fertilizers, plant protection and remove the hazard pesticides in the field of plant biochemistry. Citrus peel and tomato pomaces materials are nano-management turning into valuable pharmaceutical applications by our group^26,29^. Herein, Lemon peel Essential oil was reformulated into nanoform as potential insecticide against black cut warm. The nano-characterizations using TEM, FTIR and zeta potential indicate that our product was in nanoform. The LPEO-NPs displayed nano characteristics, when the size was less than 50 nm, and the charge was − 11.5 ± 0.9 mV. As well as the electron microscope images TEM showed the new shape of the LPEO-NPs, these nano-properties have enhanced the efficiency of the LPEO.

The finding showed that LPEO caused different levels of effects on biological parameters of *A. ipsilon* according to its concentration and formulation. Larval death was the first indicator of the insecticidal efficacy of (LPEO) and (LPEO-NPs). The results showed that, maximum percentage mortality was recorded at the high concentrations of LPEO and LPEO-NPs. The nano-emulsion of Purslane Oil exhibited 85.00% larval mortality of *A. ipsilon*^30^.

The tested concentrations significantly prolonged the duration of larvae after treated by (LPEO) and (LPEO-NPs) when compared to the control. These results agreed with the previously reported results obtained by^31^ who reported that the dehydrated f *Piper auritum*'s ethanolic extract affected the survival of *Spodoptera frugiperda* by extending the larval and pupal stages and shortening the duration of the adult stage, with the strength of the effect depending on the extract's concentration. A slight significance in larval duration elongation treated with LC_15_ and LC_50_ of Lemongrass essential oil compared with control was reported by^32^. Similarly, bulk, nano-emulsion and loaded nano-emulsion of neem oil led to larval duration elongation to 22.71, 24.40 and 24.28 days, respectively compared to control 18.82 days^33^. Similar findings confirmed that the larval duration of *A. ipsilon* was 22.61 and 24.30 days for purslane oil bulk and its nano-emulsion, respectively compared to 18.72 days in control^30^. In addition, the pupal duration of all tested concentrations was longer than control. Similar data expressed by^30^ indicated that pupal duration of *A. ipsilon* was prolonged 10.00 days compared to control (). All tested oil caused a reduction in the percentage adult emergence. These results were also agreed with previous obtained data reported by^34^ who found that Nano-formulated Jojoba oil (0.1%) caused 15.30% reduction for adult emergence. Number of emerged malformed individuals was added to the number of normal ones which explains the high percentage of adult emergence (63.33%) at concentration 25 mg/ml of LPEO. Whereas higher concentration resulted in high mortality.

Despite all surviving larvae in high concentrations pupated, the percentage of pupal mortality was lower than in the lower concentrations. In agreement with work reported by^31^, the pupal mortality of *Spodoptera frugiperda* was higher in all treatments of the larvae exposed to the LC_35_ of ethanolic extract of *Piper auritum* than LC_50_ and LC_56_. Additionally, nano-formulation using PEG of citrus peel essential oil extracted from different species including orange peel and lemon exhibited lethal activity against tomato *T. absoluta* have been reported by^21^.

The overall obtained data confirms that pupal and adult malformation% were significantly increased due to larva LPEO treatment. However, LPEO-NP slightly enhances pupal and adult malformation% compared to control. In comparing to respective controls, the results confirm that, the tested EOLP had a significant effect on the percentage of pupal and adult malformation where when larva treated with nano oil lead to raise the malformation. In general, morphological malformations occurred in response to a lack in chitin synthesis therefore inhibiting the process of ecdysis. Our findings point to an inhibition role of LPEO and LPEO-NP in chitin synthesis. In this context, Similar explanation was reported by^33,35^

who reported that the combination garlic and mint oil has a particular mode of action which interferes with the rate of deposition chitin. The newly formed cuticle become less hard, so it cannot stand the internal pressure during the molting and thus resulting in an inability to get rid of the exuviae and finally in death. Also, Liburd et al., 2000 confirmed that, the inhibition of chitinase activity can be explained by the properties of the mint oil as a growth regulator which leads to death, mostly because the insects cannot get rid of the exuviae. In this context, Amin et al. 2019 reported that, the effects of the oils on pests may attribute to their effect on pest neuro endocrine system and juvenile hormone leading to hormonal imbalance causing deformation. Basedow et al.,2012 mentioned that the treatment of beeswax with Neem Azal-T/S resulted in abnormal development and death of larval and pupal stages of greater wax moth.

Also, it has been reported that the 2nd larval instar of *A. ipsilon* was treated with different formulations of purslane oil caused prolongation in pupal duration, increasing the pupal mortality% as well as pupae malformation% by^30^. Citrus species are enriched in their contents of secondary metabolites, including polyphenolics, flavonoids, alkaloids and terpenoids^36^. The existence of terpenoids and flavonoids as a common phytochemical component in citrus extracts explainig their potential role in the toxicity against mosquitoes^37^. The insecticidal activity of *Cymbopogon citratus* (Lemongrass) is attributed to the action of major constituents citral and limonene^38,39^. The previous results showed that LPEO and LPEO-NP have potential toxic effect on different stages of the cutworm. Moreover, lemon peel essential oil nano-formulation enhances insecticidal toxicity. Another, results obtained by^40^ exhibited that insecticidal toxicity of cinnamon essential oil was increased by nanoencapsulation system using β-cyclodextrin/Gum Arabic (βCD/GA) inclusion. accordingly, the protection activity of Citronella Essential Oil (CEO) against *Spodoptera littoralis* was enhanced after its incorporation into the different chitosan nano-systems^40^. larvae of *Rhynchophorus ferrugineus* was more affected with the nano oils than the bulk^40,41^. The overall obtained results of this study concluded that, plant-derived essential oils can be used as a promising alternative pest management. Lemon Peel waste is hopeful as a natural biopesticide against the cutworm, *A. ipsilon* larvae. Furthermore, the application of Lemon Peel Essential Oil in nano form led to enhance its insecticidal toxicity.
